# Transient Coatings from Nanoparticles Achieving Broad-Spectrum and High Antimicrobial Performance

**DOI:** 10.3390/ph16060816

**Published:** 2023-05-30

**Authors:** Rachel Zaia, Giovanna M. Quinto, Livia C. S. Camargo, Rodrigo T. Ribeiro, Ana M. Carmona-Ribeiro

**Affiliations:** Biocolloids Laboratory, Departamento de Bioquímica, Instituto de Química, Universidade de São Paulo, Avenida Professor Lineu Prestes, 748, Butantan, São Paulo 05508-000, Brazil; rachelzaia@usp.br (R.Z.); gi.maruyama@usp.br (G.M.Q.); liviacestaro@usp.br (L.C.S.C.); rodrigo@iq.usp.br (R.T.R.)

**Keywords:** antimicrobial peptide, lipid bilayer fragments or disks, polyelectrolytes, pathogenic bacteria and fungus, layered nanoparticles, gramicidin nanoparticles, hydrophilic coatings from nanoparticles adhered to glass

## Abstract

Cationic and hydrophilic coatings based on casting and drying water dispersions of two different nanoparticles (NPs) onto glass are here described and evaluated for antimicrobial activity. Discoid cationic bilayer fragments (BF) surrounded by carboxy-methylcellulose (CMC) and poly (diallyl dimethyl ammonium) chloride (PDDA) NPs and spherical gramicidin D (Gr) NPs dispersed in water solution were cast onto glass coverslips and dried, forming a coating quantitatively evaluated against *Pseudomonas aeruginosa*, *Staphylococcus aureus* and *Candida albicans*. From plating and colony forming units (CFU) counting, all strains interacting for 1 h with the coatings lost viability from 10^5^ to 10^6^, to zero CFU, at two sets of Gr and PDDA doses: 4.6 and 25 μg, respectively, or, 0.94 and 5 μg, respectively. Combinations produced broad spectrum, antimicrobial coatings; PDDA electrostatically attached to the microbes damaging cell walls, allowing Gr NPs interaction with the cell membrane. This concerted action promoted optimal activity at low Gr and PDDA doses. Further washing and drying of the deposited dried coatings showed that they were washed out so that antimicrobial activity was no longer present on the glass surface. Significant applications in biomedical materials can be foreseen for these transient coatings.

## 1. Introduction

Associations between antimicrobial agents have been at the forefront of novel developments in the fight against pathogens; for example, chitosan association with enzymes [1] or antimicrobial peptides [2] improves its antimicrobial and anti−adhesive properties and prevents implant-associated infections [3,4]. New antimicrobial coatings are continuously being developed with advanced technology, employing a range of substances such as polymers [5], peptides [6,7], metals [8], e.g., copper [9] or silver [10], and metal oxides [11], e.g., zinc oxide [12,13] and titanium dioxide [14]. Other examples are polymer-based composites with specific surface chemistry and topography [5], carbon−based materials avoiding microbial adhesion such as graphene and other pathogen repellent coatings [15], contact−killing and drug-releasing systems [16,17,18], physically switchable antimicrobial surfaces and coatings depending on temperature−, light−, electric− or magnetic−field−based surface triggering [19], simvastatin−hydroxyapatite coatings on titanium alloys preventing implant−associated infections and improving bone formation [20], hydrogel coatings for drug delivery, tissue engineering, wound dressings and implants [21,22] and xerogels of porous materials comprised of dried, cross-linked polymeric networks loaded with antimicrobials [23].

Among the self−assembled antimicrobial coatings, there are interesting constructs with improved activity depicted from reduced doses of the active compounds [24,25,26]. Furthermore, self−assembled nanomaterials can be produced at low cost, and owing to their ability to self−organize, complex multifunctional structures can be tailored for their specific applications [27,28,29,30]. They have served as wound dressings [27], carriers for brain-derived neurotrophic factor for neuronal regeneration [31], growth factor delivery systems for healing chronic wounds [27], carriers for antimicrobials such as arginine antimicrobial peptide [32] or gentamicin [33], or as the antimicrobial agent themselves, such as antimicrobial bilayer fragments [34,35], bilayer−covered [36] or multilayered antimicrobial nanoparticles [37], self−assembled antimicrobial peptides [28,30], functionalized inorganic or organic polymeric materials such as chitosan and its quaternary ammonium derivatives [38], hybrid coatings of biocompatible polymer and quaternary ammonium compounds [17], and hydrogels [22]. 

**Figure 1:**
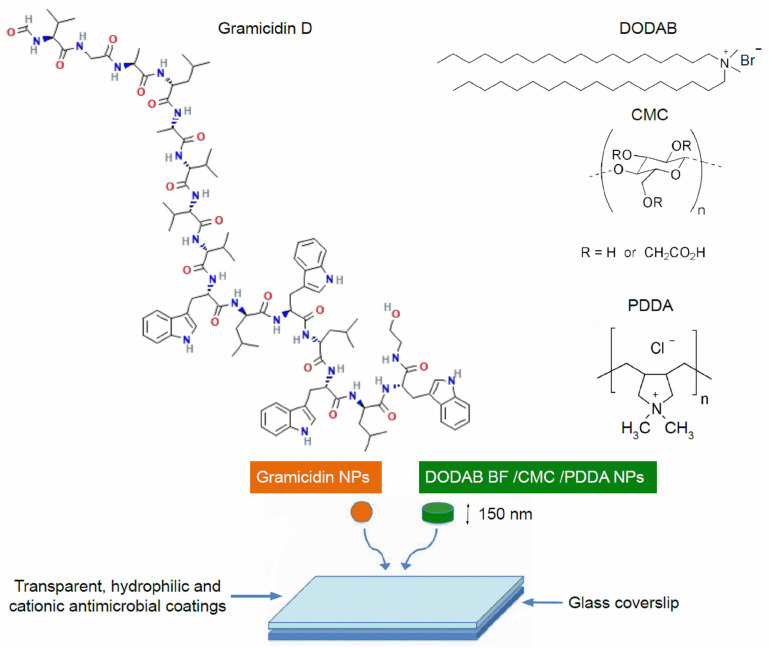
Schematic diagram of the current development of transient antimicrobial coatings on glass based on casting and drying of gramicidin (Gr) nanoparticles (NPs) [30] and self−assembled discoidal NPs [41] both in water dispersions (0.264 M D-glucose isotonic solution). The layer−by−layer discoid assemblies of dioctadecyldimethylammonium bromide bilayer fragments (DODAB BF) were covered by consecutive layers of carboxymethylcellulose (CMC) and poly diallyldimethylammonium chloride (PDDA) yielding the DODAB BF/CMC/PDDA disks in water dispersion [37]. Scanning electron micrographs for the Gr NPs and discoid NPs are available from references [30,41], respectively.

Supramolecular assemblies driven by weak, non−covalent intermolecular interactions are ubiquitous in nature and carry the potential to facilitate disassembly, a property of importance in many physiological and biological events; antimicrobial biomimetics has been providing many instances of useful assemblies fully capable of disassembling and acting in an independent but complementary manner to overcome microbial resistance to pathogens [25,30,35,39,40,41,42,43,44,45]. A huge variety of components is possible, imparting distinct and complementary antimicrobial activities to the whole assembly. Recently, we described the broad and potent antimicrobial activity of water dispersions of gramicidin (Gr) nanoparticles (NPs) and the water−soluble, hydrophilic polymer poly (diallyl dimethyl ammonium chloride) (PDDA) [30]. In this work, we construct microbicidal transient coatings based on co−deposition of cationic antimicrobial NPs [37,41] and Gr NPs [30] onto glass coverslips as substrates. The cationic antimicrobial NPs were formed layer−by−layer from cationic bilayer fragments (BF) of dioctadecyldimethyl ammonium bromide (DODAB) consecutively surrounded by carboxy−methyl cellulose (CMC) and poly (diallyl dimethyl ammonium chloride) (PDDA) layers (DODAB BF/CMC/PDDA NPs) in water dispersion. These water dispersions effectively kill Gram-negative bacteria but exhibit low activity against Gram-positive bacteria and fungus [37,40,41]. In contrast, the Gr NPs in water dispersion display activity against Gram-positive bacteria and fungus; further adding PDDA to the Gr NPs in water dispersion reduced both Gr and PDDA required doses for complete loss of viability [30]. Here, coatings from DODAB BF/CMC/PDDA NPs and Gr NPs dispersions in water were obtained by casting and drying them onto glass coverslips. Evaluation of the coatings against three different pathogens (*Pseudomonas aeruginosa*, *Staphylococcus aureus* and *Candida albicans*) from plating and colony forming units (CFU) counting yielded complete loss of viability for the three strains tested. The graphical abstract (Figure 1) summarizes how the procedure of casting and drying water dispersions of Gr spherical NPs [30] mixed with discoid DODAB BF/CMC/PDDA NPs [37,41] onto glass coverslips yielded the transparent, hydrophilic, cationic and antimicrobial hybrid coatings here described. Therefore, the concept of designing antimicrobial coatings from nanoparticles transiently providing broad and high antimicrobial activity is demonstrated in this work, opening novel possibilities for different supports used in biomedical devices such as prostheses, catheters, implants, wound dressings and others.

## 2. Results

### 2.1. Preparation and Characterization of Coatings Based on Gramicidin and DODAB BF/CMC/PDDA Nanoparticles

Coatings casted on glass coverslips from water dispersion of nanoparticles included the cationic bilayer fragments comprised of DODAB surrounded by an anionic CMC layer further adsorbing an outer cationic PDDA layer [46] and gramicidin D NPs [30]. After casting, the dispersions were dried, yielding transparent, gelatinous and hydrophilic coatings as seen from wettability determined as contact angles (θ_A_) on Table 1. Using 0.264 M D-glucose in the nanoparticles, dispersion was associated with transparency of the coatings after drying. Changing the nanoparticles’ medium to pure water also changed the macroscopic features of the coatings obtained after drying; they became more whitish. Thus, D-glucose in the nanoparticle dispersions contributed to the transparent character of the coatings. Under air, the attachment of the coatings onto the glass coverslip was strong, making it impossible to detach films from the glass. This situation contrasted with the coating’s behavior in water solutions where they could easily be disassembled from glass as described in Section 2.3.

**Figure 2:**
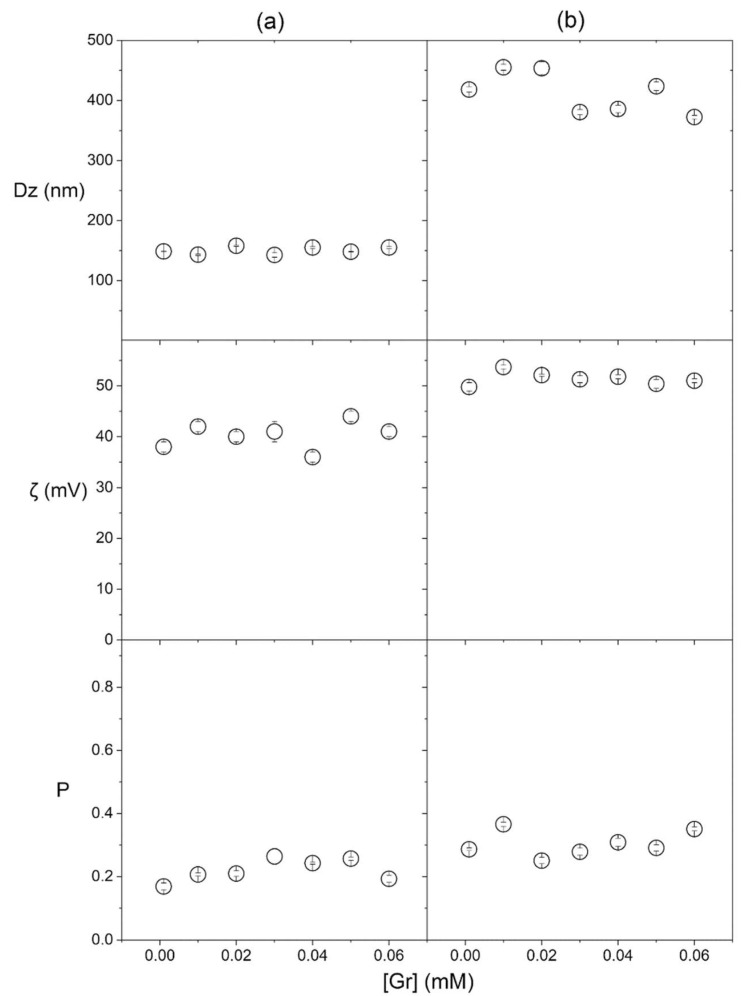
Effect of gramicidin concentration in Gr NPs on properties of small particles (SP) (**a**), or large particles (LP) (**b**). Properties were the hydrodynamic diameter (Dz), the zeta potential (ζ) and the polydispersity (P). Compositions for SP, LP or Gr NPs, are in Table 1.

The composition of nanoparticles used to obtain the coatings is described in Table 1. Five different compositions yielded coatings obtained from small particles (SP), SP and gramicidin nanoparticles (SP/Gr NPs), large particles (LP), LP and Gr NPs (LP/Gr NPs), and Gr NPs (Table 1). The physical properties of the NPs in water dispersion are in Table 1. Mean hydrodynamic diameter (Dz), polydispersity (P) and zeta−potential (ζ) determinations were in agreement with previously published data for SP, LP and Gr NPs [30,37]. For the SP/Gr NPs and the LP/Gr NPs, the physical properties were determined in this work, showing that the presence of Gr NPs did not affect Dz, P or ζ of the mixed dispersions (Table 1). Within the limits of the experimental error, the comparison between SP physical properties with or without Gr NPs showed that there was practically no change in physical properties of SP due to the presence of Gr NPs (Figure 2a; Table 1); similar results were shown for LP (Figure 2b; Table 1). 

DODAB concentration in SP was 10 times higher than Gr concentration in Gr NPs (Table 1). As a consequence, the “orange” Gr NPs were outnumbered by the “green” SP constructions based on DODAB BF; for LP at 0.5 mM DODAB mixed with Gr NPs at 0.05 mM Gr, the same occurred (Figure 1). The predominant hydrophilic nature of the coated surfaces shown by the low contact angles in Table 1 was consistent with the higher frequency of SP or LP in the coatings compared with the occurrence of Gr NPs. Gr NPs at 0.01 mM Gr and SP at 0.1 mM DODAB had the same size (Table 1), in agreement with the scanning electron micrographs (SEM) previously published for Gr NPs and SP (see Figure 1).

In water dispersion, the weak interaction between Gr NPs and PDDA polymer alone was previously reported [30]; here, one can also expect a weak interaction between Gr NPs and the outer PDDA layer of SP or LP. Gr molecules bear a net charge equal to zero; the small negative zeta-potential of −26 mV for Gr NPs was due to the anisotropy of charge distribution in Gr molecules [47] that generated slightly negative zeta-potentials for the Gr NPs. PDDA and Gr were barely attracted to each other electrostatically. In fact, Gr NPs did not change the physical properties of SP or LP (Table 1; Figure 2). As compared with the control (Gr concentration equal to zero), increasing Gr concentration did not affect Dz, P or ζ of the mixed dispersions over the 0.01–0.06 mM of Gr concentration in Gr NPs.

### 2.2. Antimicrobial Activity of the SP/Gr NPs or LP/Gr NPs Coatings against Pseudomonas aeruginosa, Candida albicans and Staphylococcus aureus

**Figure 3:**
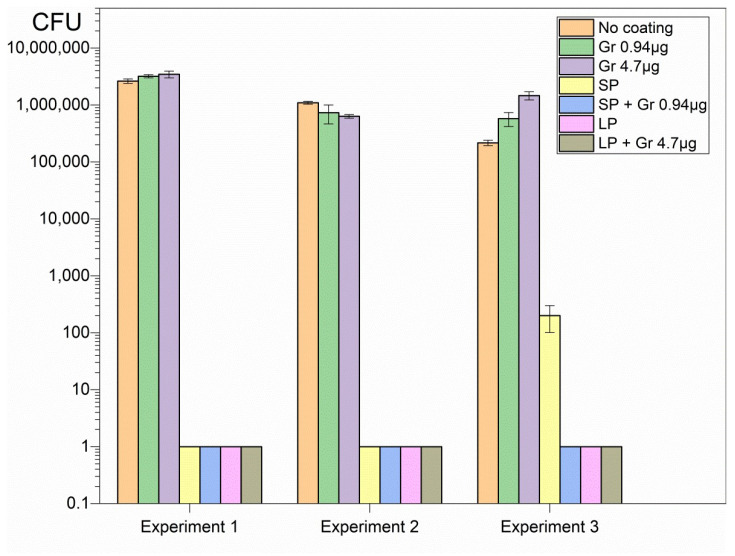
Three independent experiments showing reproducible coating’s activities against *P. aeruginosa*. Each coating was obtained from casting and drying 50 µL of Gr NPs, SP, SP/Gr NPs, LP, and LP/Gr NPs dispersions in 0.264 M D-glucose water solution. Coatings yielded the same activity independent of the time they remained on the shelf before use. Statistical analysis revealed that SP, SP/Gr NPs, LP and LP/Gr NPs coatings significantly diminished cell viability (*p* < 0.05) whereas coatings comprised of Gr NPs did not affect cell viability.

**Figure 4:**
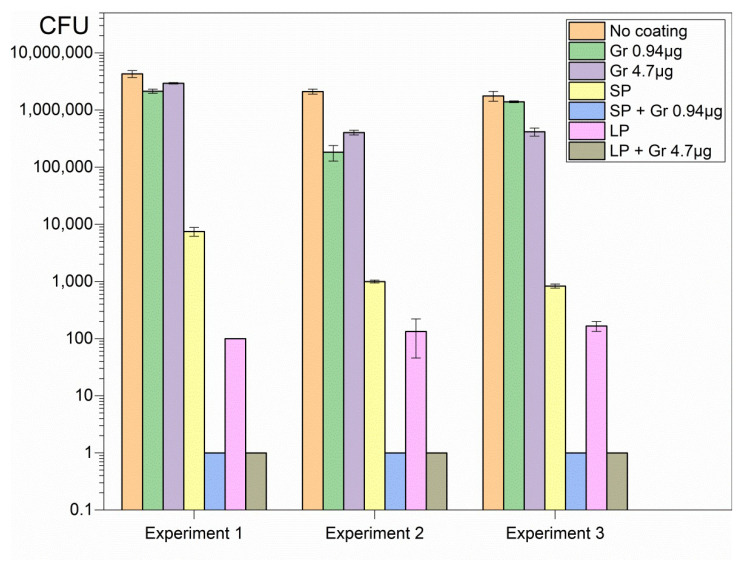
Three independent experiments showing microbicidal activity of coatings on *S. aureus*. The coatings were obtained from casting and drying 50 µL of Gr NPs, SP, SP/Gr NPs, LP, and LP/Gr NPs dispersions in 0.264 M D-glucose water solution onto glass coverslips. Statistical analysis revealed that although each type of NPs on the coatings significantly decreased cell viability (*p* < 0.05), the combinations of NPs such as SP/Gr NPs and LP/Gr NPs in the coatings brought cell viability to zero CFU count (*p* < 0.05).

**Figure 5:**
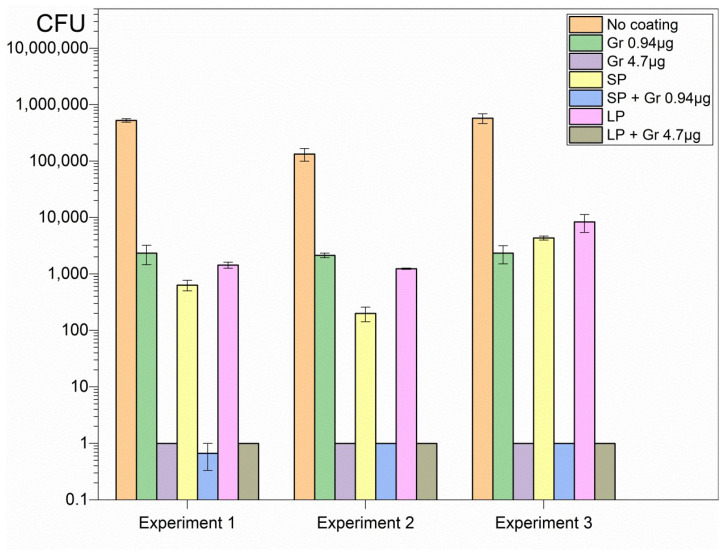
Three independent experiments showing antimicrobial activity of coatings on *C. albicans*. The coatings were obtained from casting and drying 50 µL of Gr NPs, SP, SP/Gr NPs, LP, and LP/Gr NPs dispersions in 0.264 M D—glucose water solution. Statistical analysis revealed that coatings comprised of NPs significantly decreased cell viability (*p* < 0.05).

The hybrid coatings deposited onto glass from casting and drying the dispersions revealed high activity against important representative pathogens such as *Pseudomonas aeruginosa* (Figure 3), *Staphylococcus aureus* (Figure 4), and *Candida albicans* (Figure 5).

Although SP and LP caused complete loss of cell viability against the Gram-negative bacterium *Pseudomonas aeruginosa*, neither SP nor LP could completely kill *Candida albicans* or *Staphylococcus aureus* (Figure 3, Figure 4 and Figure 5). On the other hand, Gr NPs at 0.94 or 4.7 µg Gr were completely ineffective against *Pseudomonas aeruginosa* (Figure 3); the best results were obtained with the hybrid coatings containing both SP or LP and Gr NPs, with a reduction in the CFU count from 10^6^ to 10^7^ CFU, to 0 CFU, for the three pathogens tested (Figure 3, Figure 4 and Figure 5). Gr NPs at 4.7 µg Gr were extremely effective against *C. albicans* (Figure 5) whereas SP and LP were extremely potent against *P. aeruginosa* (Figure 3). Against *S. aureus* only combinations of Gr NPs and SP or LP were completely effective, pointing to a synergistic effect of both nanoparticles (Figure 4).

Table 2 summarizes the log CFU obtained after the interaction of the three pathogens with the six different coatings. Compared with the control (pathogens interacting with bare glass coverslips), a remarkable antimicrobial activity was observed against the three pathogens tested. Despite the poor activity of Gr NPs against *S. aureus*, the combinations with SP or LP resulted in complete loss of *S. aureus* viability, revealing the power of the hybrid coatings (Table 2).

### 2.3. Antimicrobial Activity of the Coatings after Immersion in 0.264 M D-glucose Solution for 1 h

**Figure 6:**
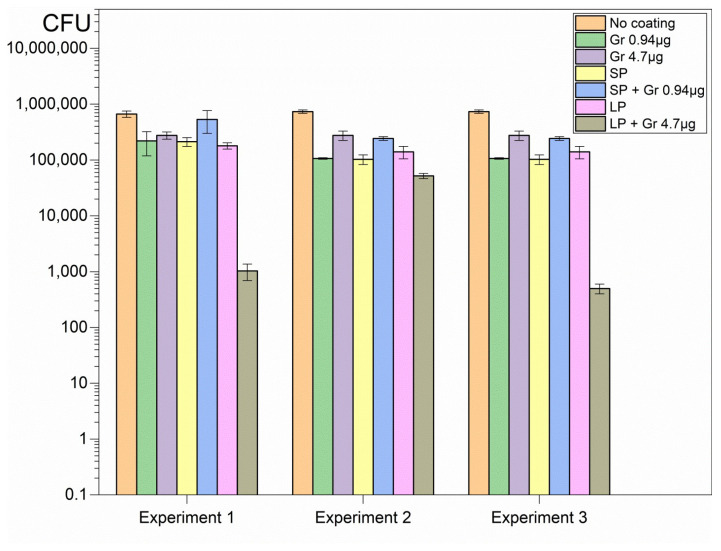
Three independent experiments showing the loss of antimicrobial activity of the coatings against *C. albicans* after immersing them in 0.264 M D-glucose aqueous solution for 1 h followed by drying.

The coatings were very stable in the air and could be used any time after casting and drying overnight, yielding reproducible antimicrobial effect independent of their age. The reproducibility of their preparation was thoroughly tested by determining the activity of the three different coatings at a given composition. It was not possible to re-use a given coating since the material was washed out as soon as the coatings were placed in a 0.264 M D-glucose solution, resulting in complete loss of antimicrobial activity against *C. albicans* (Figure 6). The magnitude of the washing-out obtained was dependent on the amount of material deposited onto the surface, as seen from the remaining activity against *C. albicans* for LP/Gr NPs (Figure 6).

**Figure 7:**
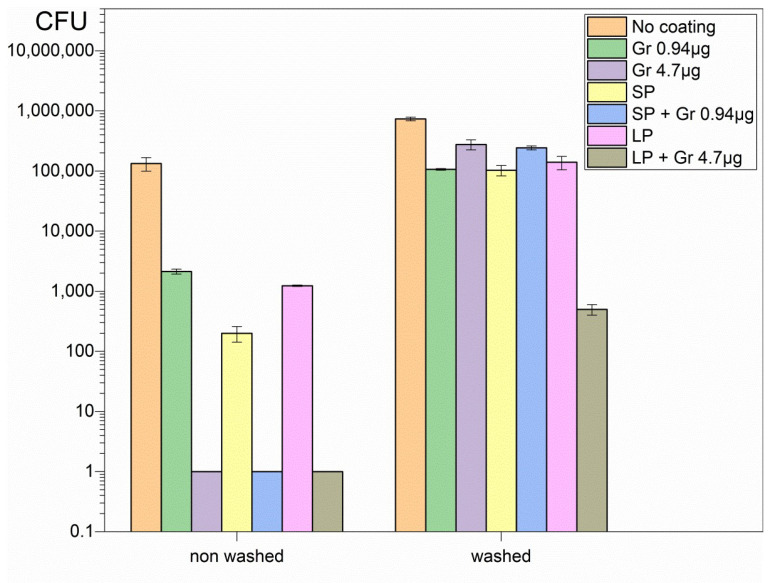
Cell viability of *C. albicans* after interacting with non-washed (Figure 5) and washed coatings (Figure 6). Due to the highly reproducible character of the non-washed and the washed coatings, only one experiment among three is shown.

The comparison between non-washed and washed coatings regarding their activity against *C. albicans* reconfirmed the complete loss of activity for the coatings with the exception of LP/Gr NPs; this coating contained the highest amount of total mass of the nanoparticles (Figure 7).

## 3. Discussion

Antimicrobial covalently bound peptide-polymer conjugates have been much explored for developing novel important materials hampering bacterial adhesion and growth at interfaces [48]. We used the advantage of peptide and polymer mixtures, that enables them to act separately since they are not bound covalently. Furthermore, the cationic character of the coatings associated with the outermost PDDA layer on SP or LP drives the interaction with oppositely charged pathogens. The transient character of the coatings implying detachment from the surface might represent an advantage for the interaction of the nanoparticles in the coating with the microbes in water dispersion. Encounters of antimicrobials and pathogens became facilitated when the coating was transferred to the bulk solution so that the cells did not need to attach to the coated surface. In vivo, this might be an advantageous asset hampering local and diffuse infection progress.

The mechanism of action of PDDA has previously been shown to involve the formation of bundles of biopolymers and PDDA, so that biomaterial from the cell wall becomes detached from this protective layer of microbes by PDDA [41,49]. PDDA damage to the cell wall facilitated the interaction of Gr NPs with the cell membrane so PDDA potentiated Gr activity. The two mechanisms of action were complementary: PDDA damaging the pathogen’s cell wall, and gramicidin forming channels in the cell membrane that destroyed the cell’s ionic balance.

Penetration of cationic polymers into microbial cells was previously reported [50,51]. Fibroblast membranes were damaged by 0.1 mg·mL^−1^ PDDA, and holes could be seen by scanning electron micrographs on the cells’ surfaces [49,52]. In this work, 0.05 mL of the water dispersions containing 0.025 mg of PDDA in LP interacted with bacteria and fungi, yielding 0.5 mg·mL^−1^, or, in SP, 0.05 mL of the water dispersions yielded 0.1 mg·mL^−1^ PDDA. All microbes were completely killed by such doses in combination with Gr NPs (Table 2). Killing the microbe by contact or mechanical perturbation of the cell wall and cell membrane is not associated with the pathogen metabolic activity and microbial resistance [53], and would be especially important for coating medical devices [54]. 

The low receding contact angle of the coatings (θ_A_) revealed their hydrophilic character (Table 1) and was possibly related to the predominant presence of PDDA at the air–water interface. Similar wettability was obtained for coatings prepared from casting and drying core/shell nanoparticles with PDDA as the shell and the hydrophobic polymer poly (methyl methacrylate) (PMMA) as the core [55]; they yielded contact angles of 9 ± 2° [44]. The presence of Gr NPs in the coatings barely affected coatings’ wettability, revealing that the self-assembly of Gr as nanoparticles in water dispersions was maintained after drying; minimization of energy for individual Gr molecules in the Gr NPs was due to favorable Gr-Gr hydrophobic intermolecular interactions; exposure of Gr hydrophobic moieties to the water phase in water dispersions could be thereby prevented by Gr self-assembly as nanoparticles and these were possibly kept as such after drying on the glass surface. 

In the coatings, Gr NPs were outnumbered by SP or LP and did not decrease the hydrophilic character of the coatings (Table 1); PDDA possibly surrounded all nanoparticles, including Gr NPs, providing bridges between them, and composed the outer layer in contact with air. The presence of PDDA at the surface of the coatings imparted positive charges to them, promoting pathogen electrostatic attraction, adsorption and killing by contact also [50].

Glass coverslips as substrates for depositing the coatings were adequate for strong coating adherence in the air driven by electrostatics since PDDA and glass have opposite charges. It was virtually impossible to detach the coatings from the glass in air. The polymeric network provided by SP or LP and Gr NPs was transparent and had a gelatinous aspect, with the Gr NPs in the fibrous net. The interaction of the coatings with the microbes for 1 h successfully promoted complete loss of microbial viability for the three important representative pathogens tested at reduced final doses of Gr and PDDA as discussed next (Figure 3, Figure 4 and Figure 5; Table 2). 

Glass coverslips are certainly not suitable substrates for biomedical applications, however other substrates such as surfaces of biocompatible polymers, cotton tissues for wound dressings or other materials for topical use might be further investigated as substrates for casting and drying the nanoparticles for antimicrobial therapy. In the clinic, diabetic patients often have problems such as residual tumor and wound infection after tumor resection; it is urgent to develop effective therapies to reach oncotherapy/anti-infection/promotion of wound healing combined treatment [56]. Thus, diabetic patients might benefit from nanoparticle-based coatings such as the ones described here, due to their strong and broad activity even in the presence of D-glucose. 

In vitro, coatings of Gr NPs alone displayed poor or no activity against the three pathogens at the doses tested; the most sensitive pathogen to Gr NPs only was *C. albicans* (Figure 5, Table 2); the explanation for this result may be related to the stability of Gr molecules within their aggregate; the hydrophobic effect between adjacent Gr molecules would stabilize individual Gr molecules in the Gr NPs. On the other hand, coatings constructed from DODAB BF/CMC/PDDA NPs only, without Gr NPs, were completely effective against Gram-negative bacterium *P. aeruginosa* but did not completely kill the Gram-positive bacterium *S. aureus* (Figure 4) or the fungus *C. albicans* (Figure 5; Table 2). 

Combining both NPs in the same coating led to complete loss of viability for the three pathogens tested in a concentration dependent-manner; the initial CFU count was reduced by 5–7 logs for the three representative microbes (Figure 3, Figure 4 and Figure 5; Table 2). The mechanism of action of each type of nanoparticle possibly accounted for this result. The transient nature of the coatings may not hamper further applications since the antimicrobial effect is often required for a short time. Furthermore, the ephemerous attachment of the coatings to the surface in water may contribute in vivo for diffusion and clearance of the antimicrobial agents and other components of the coatings. The trend of diluting PDDA and Gr by diffusion to the outer medium may even be desirable for reducing their local cytotoxicity and expanding their antimicrobial effects in the surroundings.

At last, one needs to consider that the importance of Gr goes beyond its activity against microbes. Its inhibition of cancer growth has recently been disclosed in the literature [57,58]. In addition to disrupting the ion concentration gradients of the plasma membrane, Gr also localized in the mitochondria and depolarized the inner mitochondrial membrane reducing the proton gradient and inhibiting ATP synthesis; there was mitophagy and inhibition of cancer cell growth [59]. The self-assembly of antimicrobial peptides in general [60,61], and Gr in particular [30], as well as their interactions with other assemblies able to impart desirable additional properties and activities as shown in this work, will be important in strategic areas of biomedical research.

We plan to evaluate cytotoxicity for the coatings against mammalian cells in culture in another research project. For the moment, we can comment on the dose-dependent toxicity of PDDA from references [30,52]. At 0.01 mg/mL PDDA, cell viability of fibroblasts and macrophages in culture remained high but above this dose, cell viability dropped to about 10%. Gr NPs still need further evaluation against mammalian cells.

Further testing of substrates replacing the glass coverslips are being performed in our lab, aiming at more realistic applications in clinics. In the present work, we only proved the concept that antimicrobial nanoparticles can be used to produce efficient but eventually transient antimicrobial coatings. 

## 4. Materials and Methods

### 4.1. Materials

Dioctadecyldimethyl ammonium bromide (DODAB) 99.9% pure was obtained from Sigma Co. (St. Louis, MO, U.S.A.). Carboxy-methylcellulose sodium salt (CMC) with a nominal mean degree of substitution (DS) of 0.60−0.95 was purchased from Fluka (Sigma-Aldrich, Steinheim, Germany) and poly (diallyl dimethyl ammonium chloride) (PDDA) 35% *w*/*v* low molecular weight (<100,000) was obtained from Sigma (Steinheim, Germany). The peptide mixture named gramicidin D (peptides A, B and C, consisting mostly of Gr A), chloroform, ethanol, 2,2,2-trifluoroethanol (TFE), and agar Mueller–Hinton (MHA) were from Sigma-Aldrich (St. Louis, MO, USA).

### 4.2. Preparation of DODAB Bilayer Fragments (BF) Dispersion in Water Solution

DODAB (32 mg) was dispersed in 0.264 M D-glucose water solution (25 mL) using a titanium macrotip probe powered by ultrasound at a nominal output of 90 W (20 min, 70 °C). Thereafter, the dispersion was centrifuged (60 min, 10,000× *g*, 4 °C) in order to precipitate titanium ejected from the macrotip, and the supernatant was collected for further use. This procedure dispersed the amphiphile powder in an aqueous solution using a high-energy input, which not only produced bilayer vesicles but also disrupted these vesicles, thereby generating open bilayer fragments (BF). DODAB concentration was determined by bromide microtitration [62] and adjusted to 2 mM.

### 4.3. Preparation of DODAB BF/CMC/PDDA Small (SP) and Large Nanoparticles (LP) in Water Dispersions

Stock solutions of CMC and PDDA were prepared in isotonic D-glucose 0.264 M aqueous solution at 2 and 20 mg/mL, respectively. The procedure for obtaining the SP and LP nanoparticles was previously described [37]. Briefly, CMC stock solution was added to the DODAB BF dispersion and the mixture interacted for 20 min before adding the PDDA solution for another round of interaction over 20 min. Thereafter, the DODAB BF/CMC/PDDA nanoparticles were ready for dynamic light-scattering (DLS) characterization and determination of z-average diameter (Dz), zeta-potential (ζ) and polydispersity (P) [63]. SP dispersions were obtained from final DODAB, CMC, and PDDA concentrations equal to 0.063, 0.100, and 0.100 mg/mL, respectively. LP dispersions were obtained from DODAB, CMC, and PDDA final concentrations equal to 0.315, 0.500, and 0.500 mg/mL, respectively. One should notice that 0.063 mg/mL DODAB is equivalent to 0.1 mM, and 0.315 mg/mL DODAB is equivalent to 0.5 mM DODAB.

### 4.4. Preparation of DODAB BF/CMC/PDDA Small (SP) and Large Nanoparticles (LP) in Water Dispersions Combined with Gr nanoparticles (Gr NPs)

Aliquots of a 5.0 mM gramicidin D stock solution in trifluoroethanol (TFE) once added to water dispersions yielded the Gr nanoparticles (Gr NPs), as previously described [30]. Here, the Gr NPs were obtained in previously prepared water dispersions of 0.264 M D-glucose and mixed with SP or LP also prepared in 0.264 M D-glucose; the physical properties (Dz, ζ, and P) of the combined dispersions were determined by DLS as a function of the final Gr concentration. Details of preparation and characterization for SP or LP were given on Table 1, evidencing that they were comparable to the ones previously described [37]. Gr NPs [30], SP and LP stabilities [37] were previously described as high, whereas their combinations remained stable over one week without visible precipitates. 

### 4.5. Determination of Particle Size, Zeta-Potential, and Polydispersity of SP, LP, SP/Gr NP and LP/Gr NP Dispersions by Dynamic Light Scattering (DLS)

Size, zeta-potential and polydispersity were determined by means of a ZetaPlus Zeta—Potential Analyzer (Brookhaven Instruments Corporation, Holtsville, NY, U.S.A.) equipped with a 677 nm laser and dynamic light scattering at 90° for particle sizing. Basically, Dz and the particle diffusion coefficient (D) are related by the Stokes–Einstein equation, Dz = kT/(3πηD), where k is the Boltzmann’s constant, T is temperature in Kelvin, and η is the medium viscosity. The software in the apparatus was the non-negatively constrained least squares (NNLS) for multimodal size distributions [63]. Size distributions allowed us to obtain polydispersities (P) derived from the width of size distributions. The z-average diameter is the mean hydrodynamic diameter Dz. 

Zeta—potentials (ζ) were determined from the electrophoretic mobility μ and Smoluchowski’s equation ζ = μη/ε, where η and ε are medium viscosity and dielectric constant, respectively. 

### 4.6. Preparation of Coatings from Casting and Drying the SP, LP, Gr NPs, SP/Gr NPs, and LP/Gr NPs Dispersions onto Glass Coverslips Followed by Visual Observation and Contact Angle Determinations

Coatings were prepared by casting 50 μL of the SP, LP, Gr NPs, SP/Gr NPs, and LP/Gr NPs dispersions onto glass coverslips. Thereafter, the films were transferred to a desiccator for drying overnight under vacuum and then observed and characterized regarding their wettability from sessile water droplets of 10 µL; droplets were then photographed for determining the advancing contact angle (θ_A_) five minutes after depositing the droplet on the films, as previously described [17,44,64].

### 4.7. Growth of Microbes and Determination of Microbial Cells Viability after a 1 h Interaction with the Coatings Obtained by Casting and Drying the SP, LP, Gr NPs, SP/Gr NPs, and LP/Gr NPs Dispersions onto Glass Coverslips

*Pseudomonas aeruginosa* PA14 [65,66] was kindly provided by Dr. Regina Lúcia Baldini and frozen in Luria–Bertani (LB) medium containing 20% glycerol; for reactivation and viability determination, the procedure described for the other strains was followed as described next. 

*Staphylococcus aureus* (ATCC 29213) or *Candida albicans* (ATCC 90028) were grown from stocks at −20 °C in appropriate solutions and reactivated separately by streaking them on Mueller–Hinton agar (MHA) plates for 18–48 h/37 °C incubation. Thereafter, some colonies were added to 0.264 M D-glucose isotonic solution and the turbidity at 625 nm of suspensions was adjusted to 0.5 of the McFarland scale. The 0.264 M D-glucose solution was used due to inactivation of cationic antimicrobials by ionic strength or other negatively charged components of the culture medium (e.g., amino acids or polysaccharides). 

For determination of cell viability, 50 μL of the cell suspensions (around 10^7^–10^8^ colony-forming units per mL, CFU.mL^−1^) were added on top of the coatings previously obtained from casting and drying 50 μL of each NPs water dispersion: the SP, LP, Gr NPs, SP/Gr NPs, and LP/Gr NPs dispersions cast and dried onto the glass coverslips. Coatings and microbes interacted for 1 h, before adding coated coverslips with the interacting microbes on top, onto an assay tube with 10 mL 0.264 M D-glucose isotonic solution. After stirring, 0.1 mL was withdrawn from the assay tube and either directly plated onto MHA agar or diluted 10 to 10^6^ times before plating 0.1 mL of each dilution. The plates were incubated (37 °C/24 h) before CFU counting. The logarithm of CFU counted was plotted against the types of coatings tested. CFU counting was taken as 1 for CFU counting equal to zero, so that log CFU could be taken as zero. The reproducibility of the experiments was determined from 3 independent experiments. Three different coatings prepared under the same conditions were evaluated for reproducibility. Coatings were very stable under air and could be used any time after casting and drying. A given coating, once used under water medium to be tested against the microbe suspensions, could not be further re-used due to detachment from the glass under water. This meant that the coatings were very stable under air and very unstable under water due to their highly hydrophilic character.

### 4.8. Stability of the Coatings after Further Washing and Drying Tested against C. albicans

Coatings were further tested for their remaining fungicidal activity after adding 10 mL of 0.264 M D-glucose solution for 1 h before drying and following the procedure above for interacting with *Candida albicans*, and determination of fungicidal activity from plating and CFU counting. These experiments were performed in triplicate to assess reproducibility and results shown for one representative experiment in comparison with the non-washed controls.

### 4.9. Statistical Analysis

All experiments were conducted in triplicate. Data were collected and the significant differences (*p* < 0.05) were assessed with the probability associated with two-tailed Student’s *t*-test. Each coating was tested in comparison with bare glass coverslips.

## Figures and Tables

**Table 1:** Composition and physical properties of hybrid coatings prepared from small (SP) and large nanoparticles (LP) in water dispersions (D-glucose 0.264 m water solution) with the addition of gramicidin nanoparticles (Gr NPs). The advancing contact angle (θ_A_) for each coating was expressed in degrees (°). The controls for coatings comprised of only one particle type are SP, LP and Gr NPs; their properties agreed with previously published data [30,37].

Dispersion	[DODAB] /mM	[CMC] /mg.mL^−1^	[PDDA] /mg.mL^−1^	[Gr] /mM	Dz /nm	P	ζ /mV	θ_A_/° Coating
SP	0.1	0.1	0.1	0	150 ± 2	0.18 ± 0.02	40 ± 2	9 ± 1
SP/Gr NPs	0.1	0.1	0.1	0.01	150 ± 5	0.22 ± 0.02	42 ± 2	13 ± 1
LP	0.5	0.5	0.5	0	420 ± 5	0.28 ± 0.02	50 ± 5	11 ± 1
LP/Gr NPs	0.5	0.5	0.5	0.05	420 ± 5	0.23 ± 0.02	50 ± 5	12 ± 1
Gr NPs				0.10	159 ± 1	0.14 ± 0.01	−26 ± 2	

**Table 2:** Effects of coatings based on nanoparticles on the viability of *P. aeruginosa*, *S. aureus* and *C. albicans* expressed as log CFU after a 1 h interaction between microbes and coatings. All coatings were named after the dispersions from which they were obtained.

Coating	Gr Mass/µg	PDDA Mass/µg		Log CFU	
			*P. aeruginosa*	*S. aureus*	*C. albicans*
Bare coverslip	-	-	6.1/6.0/5.1	6.1/6.1/6.1	5.7/5.2/5.7
Gr NPs	0.94	-	6.1/6.0/6.0	6.1/5.3/6.0	3.0/3.0/3.0
SP	-	5	0.0/0.0/2.0	3.2/3.0/3.0	2.8/2.4/3.3
SP/Gr NPs	0.94	5	0.0/0.0/0.0	0.0/0.0/0.0	0.0/0.0/0.0
Gr NPs	4.70	-	6.1/6.0/6.0	6.1/5.5/5.5	0.0/0.0/0.0
LP	-	25	0.0/0.0/0.0	2.0/2.0/2.0	3.0/3.0/2.8
LP/Gr NPs	4.70	25	0.0/0.0/0.0	0.0/0.0/0.0	0.0/0.0/0.0

## Data Availability

All research data are available in the present published article.
